# Electric Fields Have Potential to Accelerate MOF Synthesis

**DOI:** 10.1021/acscentsci.6c01019

**Published:** 2026-06-29

**Authors:** Daniel S. Parsons, Yueting Sun

**Affiliations:** † Amentum, Harwell, Oxfordshire OX11 0GD, United Kingdom; ‡ School of Engineering, 1724 University of Birmingham, Birmingham, West Midlands B15 2TT, United Kingdom

## Abstract

An applied electric field gives rise to a significant reduction in the reaction time for the synthesis of select metal−

## Graphical abstract

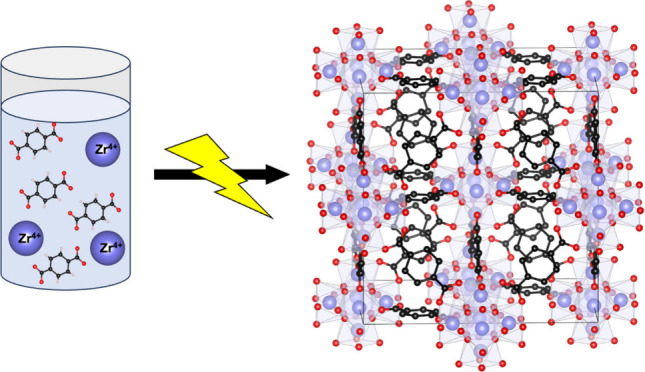

In a recent issue of *ACS Central Science*, Yan Zhou, Bing Xia, and co-workers report the use of a capacitively coupled alternating electric field (CCAEF) to accelerate the synthesis of UiO-66, a zirconium-containing metal−organic framework (MOF), and successfully extend this approach to several other MOFs containing zirconium, iron, and zinc.^1^

While many MOF materials demonstrate promising performance in a range of applications, including catalysis, drug delivery, gas separation, and storage, limited routes for cost-effective commercial synthesis have so far impeded the production and deployment of MOF technologies at industrial scales. Accordingly, technological advances that offer more energy-efficient and sustainable synthetic routes, by reducing reaction times and/or synthesis temperatures, could open economically viable routes for industrial-scale syntheses and applications.

External electric fields have been used previously to promote select chemical reactions^2,3^ and have also been used to aid crystal growth and orientation of MOF particles,^4,5^ but this report appears to be the first time they have been successfully employed in the synthesis of a MOF.^1^ Previously reported syntheses of UiO-66 typically require reaction times of 24 h at 120 °C,^6^ whereas in this study, the reaction time and temperature are reduced significantly to 25 min and 80 °C, respectively.^1^

Previously reported syntheses of UiO-66 typically require reaction times of 24 h at 120 °C, whereas in this study, the reaction time and temperature are reduced significantly to 25 min and 80 °C, respectively.

The authors report a series of experiments that systematically vary parameters to understand the impact of temperature, voltage, and current, including both alternating (AC) and direct current (DC), on the reaction time and the product generated. Ultimately, the authors report that AC is superior to DC, and present optimized parameters for the reaction temperature, applied voltage, and current. Control experiments also demonstrate that the promoting effect is solely due to the electric field and that no significant heating is caused when the field is applied to the experimental apparatus. Further control experiments suggest that the likely mechanism is accelerated conversion of the zirconium chloride starting material to the reactive intermediates, zirconium-oxo clusters (Figure 1), as evidenced by bands attributed to Zr–O bonds in the Raman spectrum.

**Figure 1:**
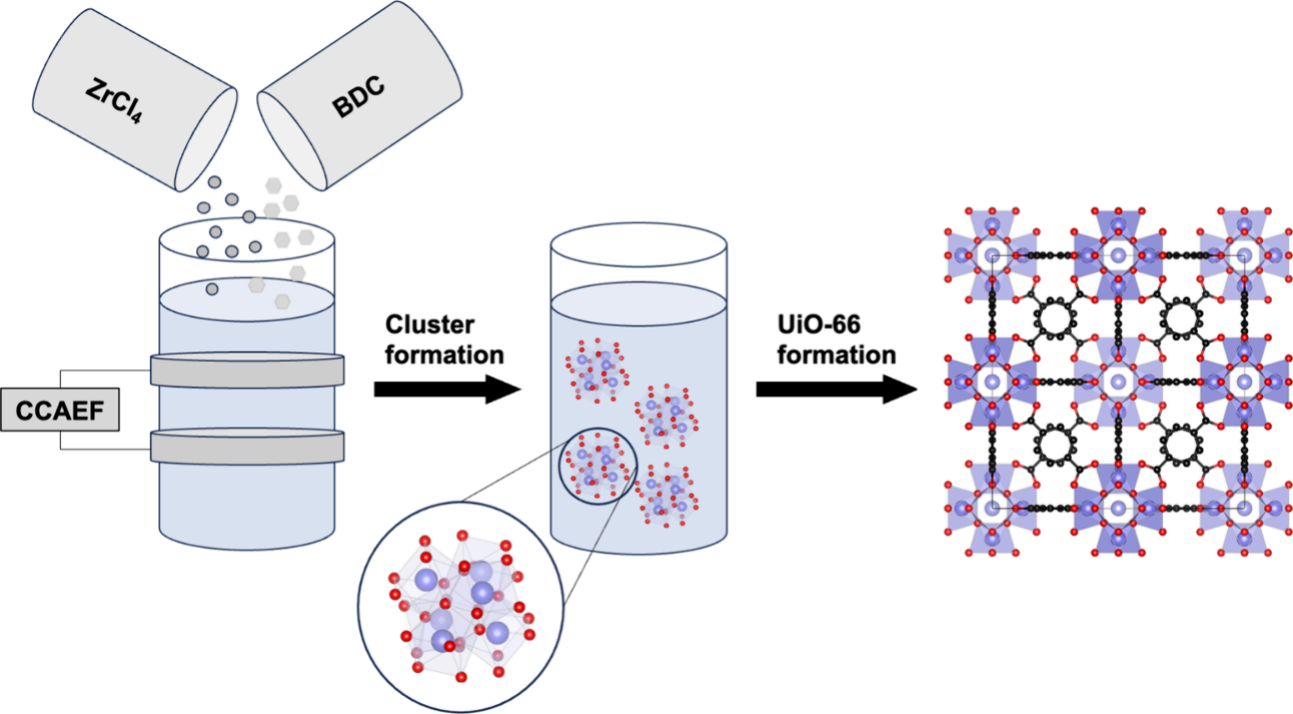
Accelerated synthesis of UiO-66 via the activation of metal precursor by CCAEF.

Further control experiments suggest that the likely mechanism is accelerated conversion of the zirconium chloride starting material to the reactive intermediates, zirconium-oxo clusters, as evidenced by bands attributed to Zr–O bonds in the Raman spectrum.

While the importance of the electric field in promoting the formation of zirconium-containing reactive intermediates has been established when this approach is applied to UiO-66, several mechanistic questions remain and warrant future investigation. The authors have optimized the voltage and current for the applied electric field, but the relationship between these parameters and their impact on the reaction is not yet understood. Future studies can probe why seemingly minor variations lead to the formation or absence of the desired product. Raman spectroscopy has identified the generation of species containing Zr–O bonds in the electric field. Future research can aim to reveal the exact nature of these species (i.e., are they tetrameric or hexameric zirconium-oxo clusters? Or are they a different zirconium-oxo species?). Identifying the intermediate species will enable a full mechanistic understanding of the promoting effect from the electric field.

In addition to UiO-66 and several other zirconium MOFs, the authors successfully demonstrate the promoting effect of the electric field in the synthesis of MOFs containing zinc (MOF-5) and iron (MIL-53 and MIL-88A), which shows the wide utility of the approach. Future research could investigate the reactive intermediates produced or the underlying mechanisms in these systems. An enhanced understanding may allow optimization of this technique and identification of other MOFs that could benefit from this approach.

In the iron-containing MOF systems assessed (MIL-88A and MIL-53), it is noted that reduction of Fe­(III) to Fe­(II) is observed. We interpret the authors’ suggested explanation for this phenomenon as the impact of the CCAEF on enhancing electron-transfer processes during the reaction. While applied electric fields can influence the favorability of redox reactions and the stabilization of particular oxidation states, the reduction of Fe­(III) to Fe­(II) in a system without an electrode contacting the solution will proceed concomitantly with the oxidation of another species in the system. It would therefore appear that the chemistry within the iron-containing systems is more complex when an external electric field is applied. Future investigations of this phenomenon would allow the mechanism for iron reduction to be elucidated.

It is worth highlighting that all zirconium-containing MOFs synthesized using the CCAEF method possessed particle sizes with diameters ca. 78 nm. Established synthetic methods for UiO-66 and UiO-66-NH_2_ typically produce larger particles with approximate diameters of 150 and 200 nm, respectively.^7,8^ The potential control over particle size afforded by this technique would also appear to be worth investigating in future work, as this may be a further benefit of the CCAEF approach beyond the significantly reduced reaction times.

The authors have demonstrated that the synthesis can be scaled up 10-fold, from initial experiments with a 10 mL total solvent volume to a total volume of 100 mL. Future work can be done to determine whether the approach works and is feasible at operational scales for commercial production which may be thousands of times greater in volume. Beyond the chemical exercise of demonstrating synthesis viability at operational scales, the feasibility of designing a suitable reactor that successfully integrates a plasma generator capable of producing a sufficient CCAEF would also need to be investigated. If both the chemical and engineering challenges of scale-up are overcome, energy consumption for the process could be much lower than for conventional synthetic routes, as the reaction time is reduced significantly along with a marked reduction in temperature. Nevertheless, the additional energy consumed by the plasma generator that generates the electric field would need to be calculated and taken into account in any quantitative comparisons on reduced energy consumption.

It is naturally too early to tell how influential this work will become, but it certainly possesses the originality of seminal work and the hallmark of disruptive technology.

Ultimately, the application of a CCAEF shows promise in reducing the energy demand, sustainability, and commercial viability of MOF synthesis in a number of systems. This novel and innovative work should serve as a springboard for future investigations to underpin the mechanism by which the electric field promotes the chemical reactivity and to determine whether this technology can be employed for more MOF systems than those studied. Some questions remain including whether this approach can be successfully scaled up and deployed industrially, a full understanding of the underlying mechanisms behind the promotion of the reactivity, and how many other significant MOFs this approach can be successfully extended to. It is naturally too early to tell how influential this work will become, but it certainly possesses the originality of seminal work and the hallmark of disruptive technology.
